# Systematic evaluation of antimicrobial food preservatives on glucose metabolism and gut microbiota in healthy mice

**DOI:** 10.1038/s41538-022-00158-y

**Published:** 2022-09-13

**Authors:** Ping Li, Ming Li, Tao Wu, Ying Song, Yan Li, Xiaochang Huang, Hui Lu, Zhenjiang Zech Xu

**Affiliations:** State Key Laboratory of Food Science and Technology, Nanchang University, No. 235 Nanjing East Road, Nanchang, 330047 China

**Keywords:** Risk factors, Health care

## Abstract

Certain antimicrobial preservatives (APs) have been shown to perturb gut microbiota. So far, it is not yet fully known that whether similar effects are observable for a more diverse set of APs. It also remains elusive if biogenic APs are superior to synthetic APs in terms of safety. To help fill these knowledge gaps, the effects of eleven commonly used synthetic and biogenic APs on the gut microbiota and glucose metabolism were evaluated in the wild-type healthy mice. Here, we found that APs induced glucose intolerance and perturbed gut microbiota, irrespective of their origin. In addition, biogenic APs are not always safer than synthetic ones. The biogenic AP nisin unexpectedly induced the most significant effects, which might be partially mediated by glucagon-like peptide 1 related glucoregulatory hormones secretion perturbation.

## Introduction

Advances in food technology have resulted in increasing food additives added intentionally to food for enhancing its taste, flavor, color, texture and nutritional value^1,2^. Antimicrobial preservatives (APs) are commonly used food additives that are chemically synthetic or biogenic (naturally extracted or biotechnological) substances. APs can prevent or inhibit spoilage of food due to fungi, bacteria, and other microorganisms and extend shelf life by reducing degradation and other unwanted reactions^3^. Several food additives have been proved to exert negative effects on the gut microbiota and therefore the host, such as emulsifier^4,5^ and artificial sweeteners^6^. Given the antibacterial properties of APs, they might also impact gut microbiota once ingested with food.

Limited studies have suggested APs can served as potential triggers of gut microbiota dysbiosis in vitro and animal models. Sodium bisulfite and sodium sulfite inhibited the growth of four species of beneficial gut bacteria in vitro^7^. Potassium sorbate was reported to change gut microbiota composition and inhibit gut microbiota metabolism of zebrafish^8^. Similarly, nitrate exposure induced gut microbiota dysbiosis and metabolism disorder in *Bufo gargarizans* tadpoles^9^. In rodent models, mixture of sodium benzoate, sodium nitrite and potassium sorbate induced dysbiosis in both wild-type and human microbiota-associated Nod_2_^−/−^ mice^10^. In addition, nitrated meat products were reported to alter gut microbiota, behavior and brain gene expression in rats^11^.

Up to now, systematic study about the impact of APs on gut microbiota has not been reported yet. And it remains elusive if biogenic APs are superior to synthetic ones in terms of safety^2^. Here, we evaluated the impact of chronic exposure of eleven commonly used APs, including both synthetic and biogenic APs, on gut microbiota in the wild-type healthy mice instead of disease mice model from genetic manipulation or diet induction, for it may be more relevant to access their effects on normal hosts. In addition, in light of the consensus on the vital role of gut microbiota in metabolism homeostasis^12^, APs-induced gut microbiota dysbiosis might further perturb host metabolism. Then, APs were further evaluated for their impacts on glucose metabolism.

## Results

### Chronic APs consumption induced glucose intolerance

**Fig. 1:**
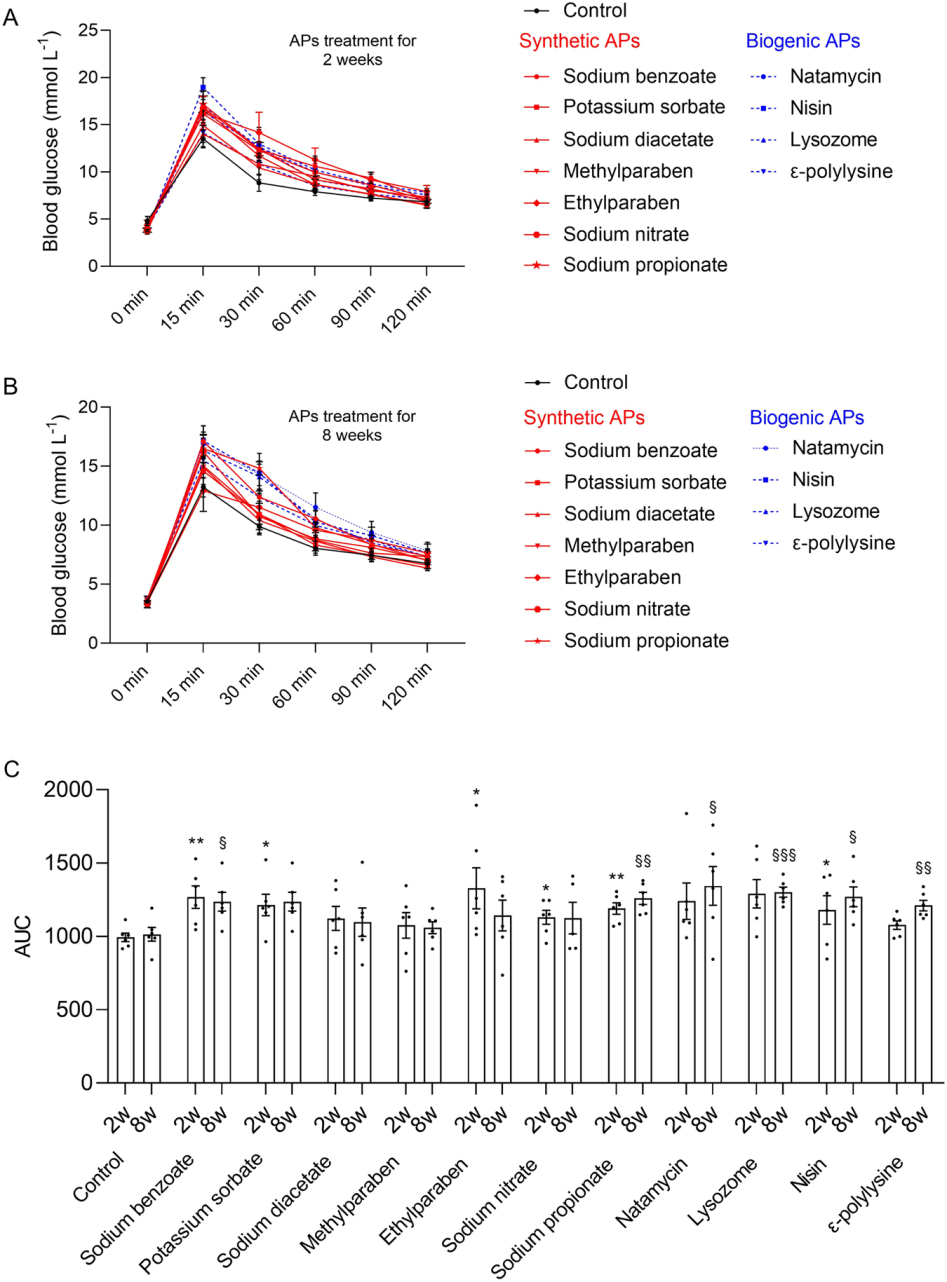
Chronic APs administration induced glucose intolerance. **A** Oral glucose tolerance test (OGTT) after 2 weeks treatment. **B** OGTT after 8 weeks treatment. **C** The 2-h blood glucose response curve of the second week (^*^P < 0.05, ^**^P < 0.01) and eighth week (^§^*P* < 0.05, ^§§^*P* < 0.01, ^§§§^*P* < 0.001)^,^ respectively. Significance was determined using two-tailed *t*-tests. The values are expressed as the means ± S.E.M., *n* = 6 per group.

Glucose tolerance test was conducted at the second and eighth weeks after treatment (Fig. 1A, B), respectively, to evaluate the time-dependent effects of APs consumption on glucose homeostasis in mice. As shown in Fig. 1C, after APs treatment for 2 weeks, five synthetic APs, namely sodium benzoate (*p* = 0.0073), potassium sorbate (*p* = 0.0191), ethylparaben (*p* = 0.0424), sodium nitrate (*p* = 0.0340), and sodium propionate (*p* = 0.0024), and biogenic AP nisin (*p* = 0.0147) showed significantly hyperglycemic effects. Then, glucose tolerance test was conducted again to determine the chronic long-term effects of APs on blood glucose level after APs treatment for 8 weeks. It showed that two synthetic APs [sodium benzoate (*p* = 0.0179) and sodium propionate (*p* = 0.0027)] and all the biogenic APs [natamycin (*p* = 0.0402), nisin (*p* = 0.0005), lysozyme (*p* = 0.0109) and ε-polylysine (*p* = 0.0070)] significantly impacted the blood glucose level. Comparing the blood glucose response between the two time points (Fig. 1C), potassium sorbate, sodium nitrate and ethylparaben just exerted transient effects on blood glucose level, while sodium benzoate and sodium propionate showed sustaining effects on blood glucose level. Surprisingly, all the biogenic APs showed time-dependent hyperglycemic effects. The results indicate that chronic exposure of APs impact glucose metabolism irrespective of synthetic or biogenic origin. And the biogenic AP nisin induces the most significant glucose intolerance.

### Chronic APs exposure altered gut microbiota

**Fig. 2:**
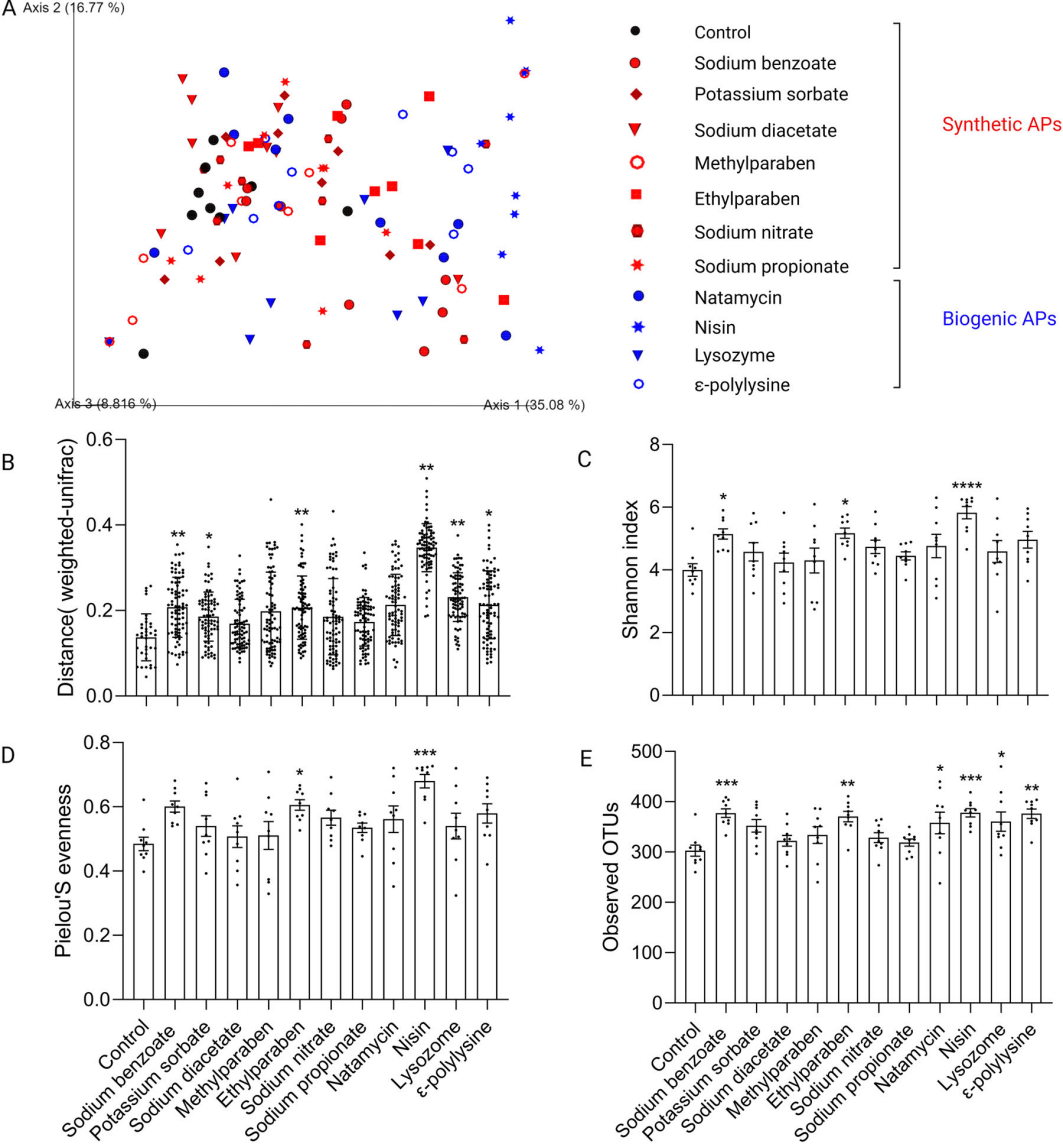
Impact of APs on gut microbiota diversity. **A** PCoA of the Weighted UniFrac distance matrix of fecal microbiota. **B** Weighted UniFrac distances between APs-treated and control microbiota was plotted and compared to the distances within control samples. Significance was determined by PERMANOVA. FDR ^*^P < 0.05, ^**^P < 0.01. **C**–**E** Alpha diversity analysis of gut microbiota between control and APs-treated groups measured with Shannon index (**C**), Pielou’s evenness index (**D**), and the number of observed OTUs (**E**). Significance was determined by ANOVA with Dunnett’s multiple comparisons test. The values are expressed as the means ± S.E.M., *n* = 9 per group. ^*^P < 0.05, ^**^P < 0.01, ^***^P < 0.001, ^****^P < 0.0001.

After APs treatment for 8 weeks, the effects of chronic APs exposure on gut microbiota were determined by 16S rDNA sequence analysis of feces samples. Investigation of beta diversity, APs-treated samples showed different level of shift from control (Fig. 2A). The Weighted Unifrac distances separating APs-treated from untreated microbiota were quantified and further analyzed by permutational multivariate analysis of variance (PERMANOVA). As shown in Fig. 2B, three synthetic APs [namely, sodium benzoate (*p* = 0.0051), potassium sorbate (*p* = 0.0440), and ethylparaben (*p* = 0.0051)] and three biogenic APs [namely, lysozyme (*p* = 0.0094), nisin (*p* = 0.0051), and ε-polylysine (*p* = 0.0124)] exerted significant impacts on gut microbiota. It indicates that APs impact the gut microbiota irrespective of their origin. Notably, the biogenic AP nisin exhibited the most significantly distinct microbiota (Fig. 2B). Moreover, nisin significantly increased alpha diversity measured with Shannon index, Pielou’s evenness index, and the number of observed OTUs (Fig. 2C–E).

**Fig. 3:**
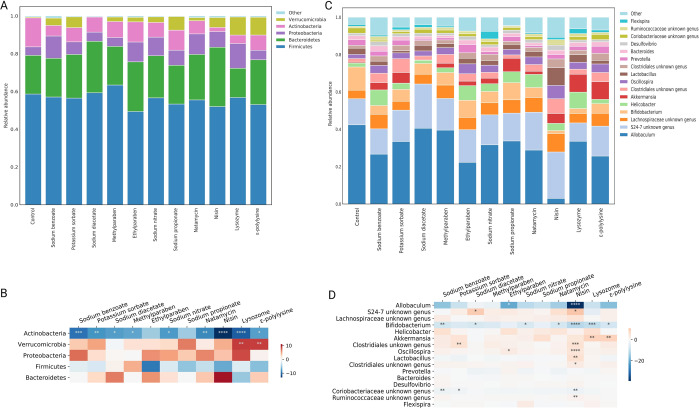
APs treatment altered microbiota composition at various taxonomic levels. **A** and **C** Microbiota composition at the phylum (**A**) and genus level (**C**). **B** and **D** Only the five more abundant phyla (**B**) and the 16 more abundant genera (**D**) were represented. Significance was determined using ANOVA corrected for multiple comparisons with a Dunnett’s test. Color coding scale indicates the relative abundance difference between individual AP and control. Grids in red indicate higher relative abundance compared with control, grids in blue indicate lower relative abundance compared with control. *n* = 9 per group. *P < 0.05, **P < 0.01, ***P < 0.001, ****P < 0.0001.

Taxonomic analysis at the phylum level revealed that *Firmicutes, Bacteroidetes, Proteobacteria, Actinobacteria* and *Verrucomicrobia* were the most abundant phyla in both control and APs-treated groups (Fig. 3A). As shown in Fig. 3B, compared with control, chronic APs treatment induced a significant reduction of *Actinobacteria*. While *Verrucomicrobia* increased in most APs-treated groups, especially lysozyme (*p* = 0.0030) and ε-polylysine (*p* = 0.0035), concurring with the previous study showing the high-level colonization of *Verrucomicrobia* in human gut following broad-spectrum antibiotic treatment^13^. In addition, the relative abundance of *Proteobacteria* was enriched by sodium benzoate, ethylparaben, sodium nitrate, sodium propionate, natamycin, nisin, and lysozyme, indicating the dysbiosis tendency of gut microbiota. At the genus level, the effect of APs on the microbiota composition was highly differential (Fig. 3C). Amongst the 16 most abundant genera (Fig. 3D), potential probiotic *Bifidobacterium* was significantly decreased by sodium benzoate (*p* = 0.0052), sodium diacetate (*p* = 0.0240), sodium nitrate (*p* = 0.0349), natamycin (*p* = 0.0139), lysozyme (*p* = 0.0002) and ε-polylysine (*p* = 0.0105). However, *Akkermansia genus* considered as next-generation beneficial microorganisms and reported to improve glucose metabolism disorder^14^, which was significantly enriched by lysozyme (*p* = 0.0030) and ε-polylysine (*p* = 0.0035). The *Coriobacteriaceae* family that can convert carbohydrates to acetic acid and lactic acid^15^ was decreased by all of the APs, especially sodium benzoate (*p* = 0.0086) and potassium sorbate (*p* = 0.0396). The opportunistic pathogen *Helicobacter* were enriched by most of the APs. In addition, APs exerted limited impact on the *Lachnospiraceae* family and genera of *Prevotella*, *Bacteroides*, *Desulfovibrio*, and *Flexispira*. Of concern is that nisin-treated group exhibited the most distinct microbiota feature with significant decrease of taxa belonging to the *Bifidobacterium* genus (*p* < 0.0001)*, Coriobacteriaceae* family (*p* = 0.0019), and *Allobaculum* genus (*p* < 0.0001). And the taxa belonging to the *Oscillospira* genus (*p* < 0.0001), *S24-7* family (*p* = 0.0197), *Clostridiales* order (*p* = 0.0003), *Ruminococcaceae* family (*p* = 0.0036), and *Lactobacillus* genus (*p* = 0.0072) were significant enriched by nisin. Given the most significant impacts on glucose metabolism and gut microbiota, we focused on nisin for the following study to explore the possible mechanism of glucose intolerance.

### Nisin induced microbial metabolites alteration

**Fig. 4:**
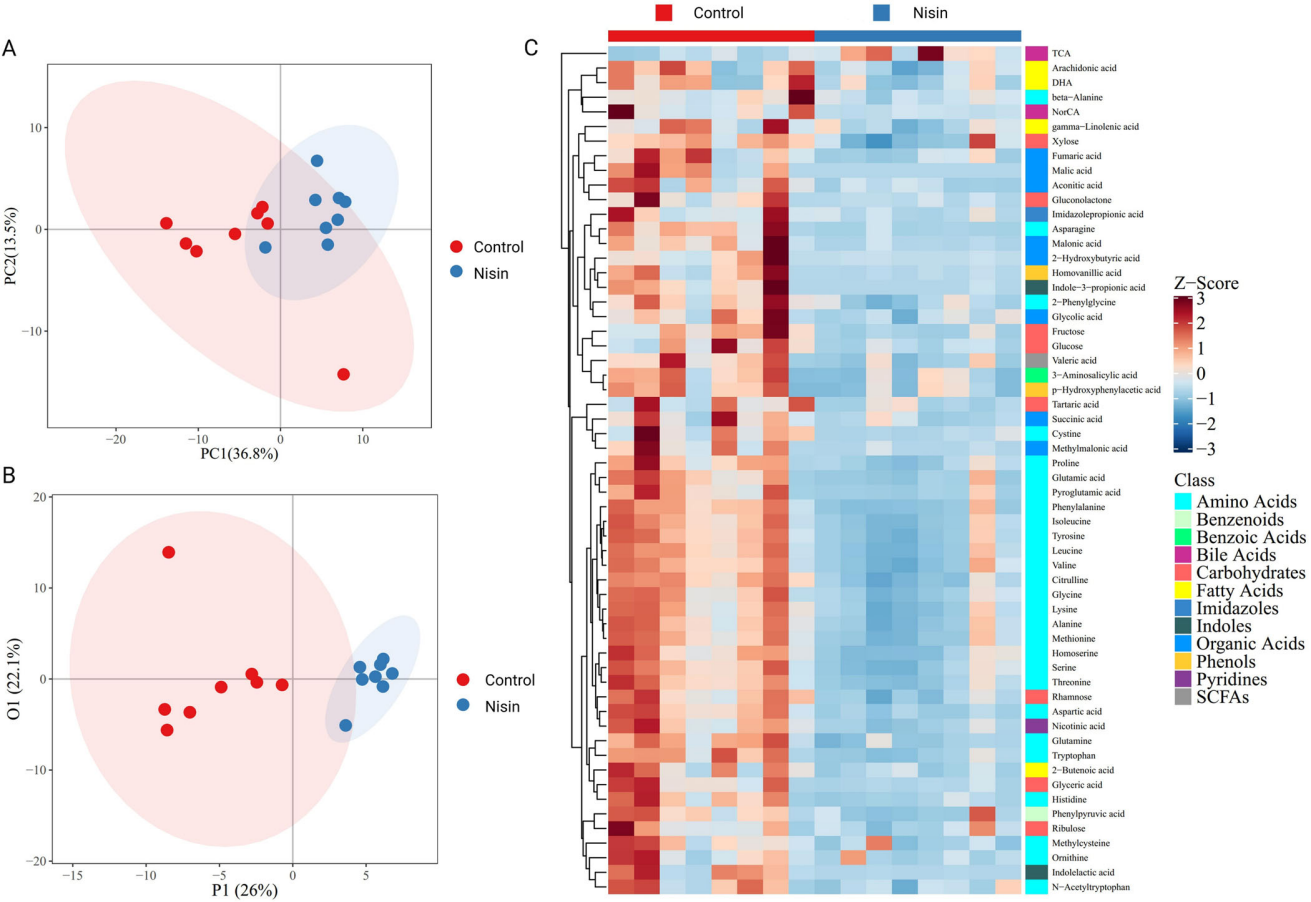
Effect of nisin on gut microbiota associated metabolism analyzed by targeted metabolomics in feces samples. **A**, **B** PCA (**A**) and OPLS-DA (**B**) indicating clear separation between control and nisin-treated samples. **C** Hierarchical clustering of heatmap displaying the 58 significantly changed metabolites between two groups. *n* = 6 per group.

Distinct clustering of metabolites was shown between control and nisin-treated samples in the principal components analysis (PCA) plot (Fig. 4A), which indicated significant alteration of metabolites induced by nisin. The data matrix was further analyzed by orthogonal partial least squares discriminant analysis (OPLS-DA). As showed in Fig. 4B, the OPLS-DA score plots clearly separated between control and nisin-treated group. The parameters of R^2^Y = 0.858 and Q^2^Y = 0.405 indicated good predictability and reliability of the OPLS-DA model.

**Fig. 5:**
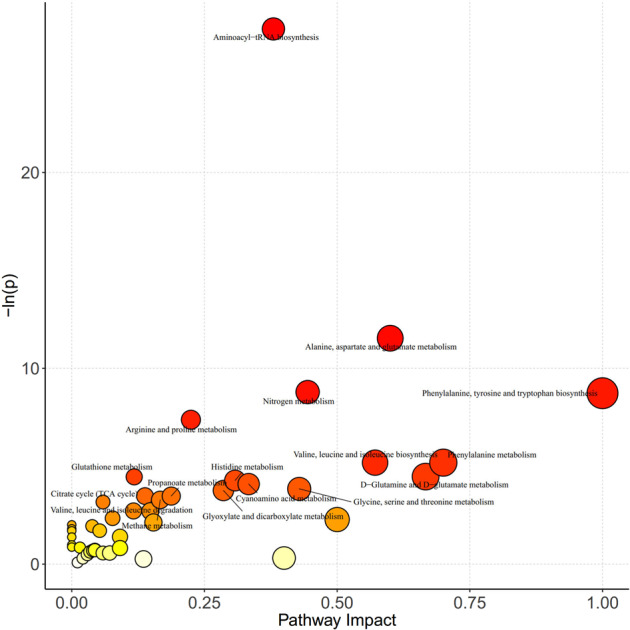
Kyoto Encyclopedia of Genes and Genomes pathway analysis of differential metabolites. *X*-axis represents the pathway impact obtained by the out-degree centrality algorithm. The size of the point is related to the pathway impact. *Y*-axis represents the negative logarithm of the *p*-value (−ln(p)) obtained by the pathway enrichment analysis. The yellow-red color change of the point is positively related to the −ln(p). The names of pathways are labeled in the graph with −ln(p) > 3.

Potential metabolic biomarkers were selected with a variable importance in the project value higher than 1.0 in the OPLS-DA and *p*-value < 0.05. There were 58 potential metabolic biomarkers significantly altered by nisin, which were visualized in a heatmap (Fig. 4C). Taurocholic acid was significantly upregulated and other 57 metabolites were significantly downregulated in the nisin-treated group, including amino acids, carbohydrates, fatty acids, organic acids, benzenoids, benzoic acids, bile acids, imidazoles, indoles, phenols, pyridines and short-chain fatty acids. Among them, amino acids were the most remarkable decreased metabolites. Kyoto Encyclopedia of Genes and Genomes pathway analysis of differential metabolites (Fig. 5) further found that metabolic pathways of phenylalanine, tyrosine and tryptophan biosynthesis, phenylalanine metabolism, D−glutamine and D−glutamate metabolism, valine, leucine and isoleucine biosynthesis, alanine, aspartate and glutamate metabolism, and aminoacyl−tRNA biosynthesis were significantly changed.

### Correlation analysis of targeted metabolomics and gut microbiota

**Fig. 6:**
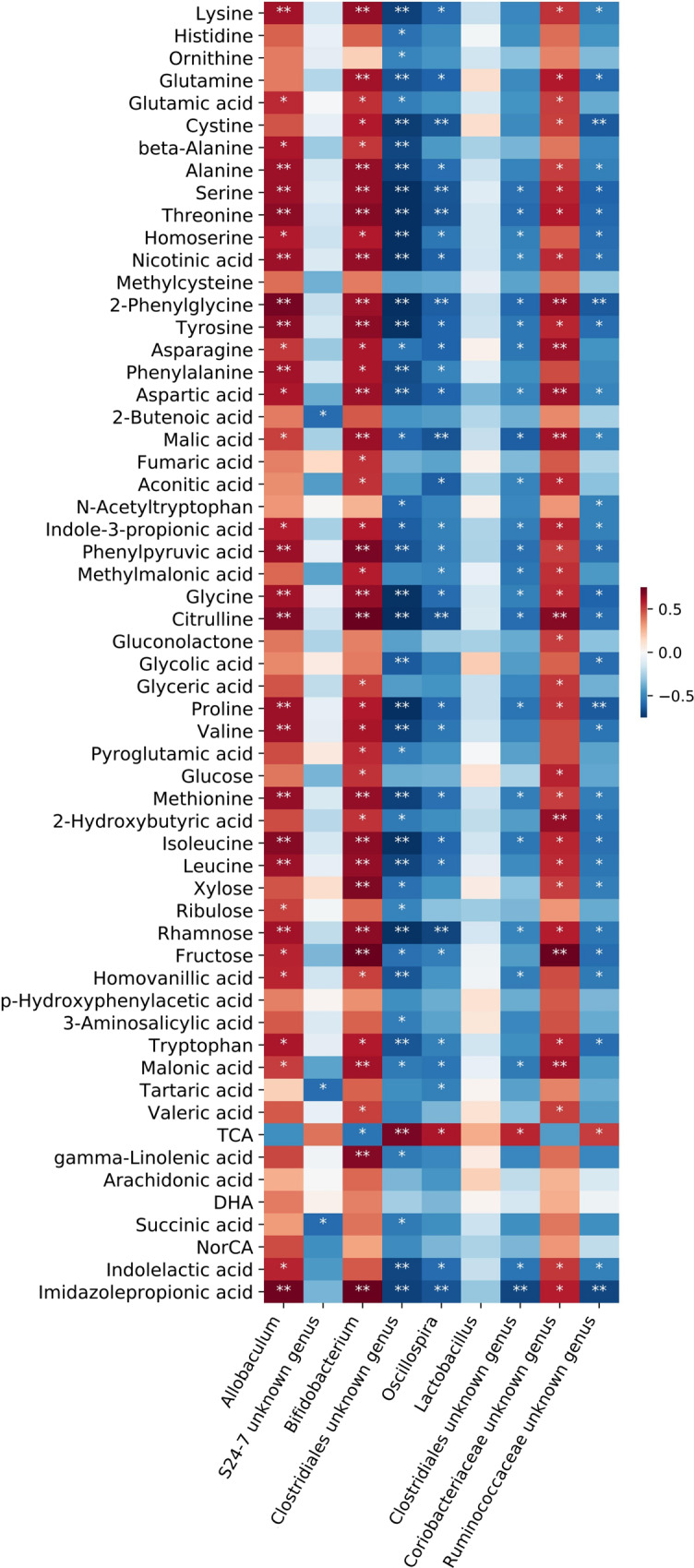
Correlation analysis of targeted metabolomics and 16S rRNA sequencing. Color coding scale indicates the correlation analysis value from heatmap, the deeper red or blue indicates higher correlation values. Grids in red indicate positive correlations, while grids in blue indicate negative correlations. *p < 0.05, **p < 0.01.

Among the top 16 genera with relative abundance, nine genera had undergone significant changes through nisin treatment compared with control (Fig. 3D). Therefore, at the genus level, spearman’s correlation analysis was used to analyze the correlation between the differential genus and metabolites. As shown in Fig. 6, *Allobaculum*, *Bifidobacterium* and *Coriobacteriaceae unknown genus* showed positive correlations and *Oscillospira*, *Clostridiales unknown genera* and *Ruminococcaceae unknown genus* showed negative correlations with all the downregulated metabolites. The only upregulated metabolite taurocholic acid showed significantly negative correlation with *Bifidobacterium* and significantly positive correlation with *Clostridiales unknown genera*, *Oscillospira*, and *Ruminococcaceae unknown genus*.

### Nisin reduced GLP-1 expression and altered glucoregulatory hormone secretion

**Fig. 7:**
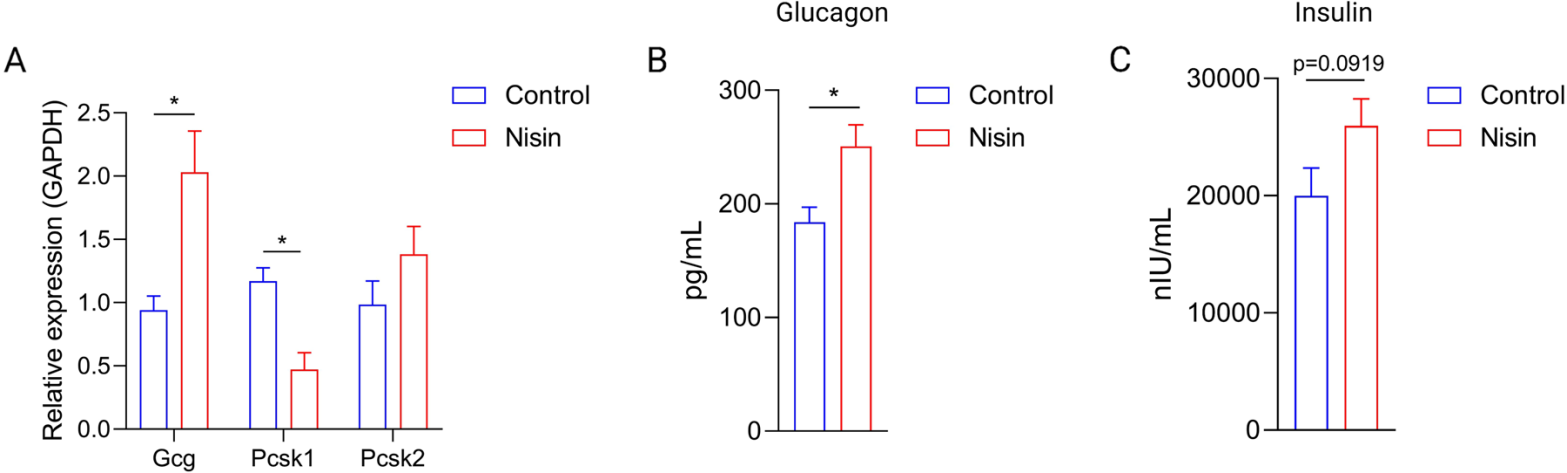
Nisin induced GLP-1 related glucoregulatory hormones secretion alteration. **A** RNA expression of Gcg, Pcsk1 and Pcsk2 (*n* = 6). **B** Glucagon level in the serum (*n* = 8). **C** Insulin level in the serum (*n* = 8). Significance was determined using two-tailed unpaired *t*-tests. The values are expressed as the means ± S.E.M. *P < 0.05.

Nisin-treated group exhibited remarkable decrease of amino acids, which might impact GLP-1 release. Therefore, we examined GLP-1 expression in the colon samples after 8 weeks treatment. Transcription of Gcg (which encodes proglucagon), prohormone convertase 1 (Pcsk1) and Pcsk2 was measured in control and nisin-treated groups. GLP-1 are produced from proglucagon through proteolytic cleavage by Pcsk1^16^. As shown in Fig. 7A, significantly lower expression of Pcsk1 (*p* = 0.0149) were found in nisin-treated mice.

GLP-1 regulates glucose metabolism in great part through stimulation of nutrient-induced insulin release and by reducing glucagon secretion^17,18^. The, insulin and glucagon in the serum were further tested by ELISA after treatment. As shown in Fig. 7B, C, glucagon significantly increased in the nisin-treated group (*p* = 0.0123). In addition, nisin treatment increased insulin secretion (*p* = 0.0919). The glucoregulatory hormones secretion perturbation might mediate nisin induced glucose metabolism disorder.

## Discussion

Food additive sweeteners have been reported to induce glucose intolerance in mice^6^. However, it is still unresolved whether the commonly used APs are a risk factor in this risk. In this study, both synthetic and biogenic APs induced glucose intolerance in healthy mice. Therefore, the chronic exposure to these APs alone or in combination may be a concern, especially for the population with glucose metabolic disorders status. For evaluating the impacts of APs on the gut microbiota, APs were added to normal diet of mice at three times the acceptable daily intake (ADI) level, which simulated the high-level APs exposure consumers by referring the dietary exposure level of food additives in the population^19^. APs exhibited highly heterogeneous impact on gut microbiota, which was irrespective of origin. However, given the both positively and negatively influence of altered genus on glucose homeostasis, further studies such as fecal microbiota transplant experiment need be conducted to validate the effect of APs induced gut microbiota alteration on glucose metabolism.

Nisin is an antimicrobial peptide produced by *Lactococcus lactis subsp. lactis*^20^. It is reported that nisin can be digested by pancreatin in the small intestine, thus may not reach the lower gastrointestinal tract (GIT) and exert little impact on gut microbiota^21^. However, nisin inhibited *Clostridium difficile* and temporarily changed microbiota in a model of human colon in vitro^22^. In addition, intact and biologically active nisin were detected in the feces of mice after oral delivery of nisin in resistant starch based matrices^23^. These studies indicated that nisin can be delivered to the lower GIT owning to the protection from food component, thus exerting impacts on the lower GIT microbiota.

Nisin induced gut microbiota alteration and remarkably downregulated amino acids, concurring with the previous reports that gut microbiota influences the free amino acid distribution in the intestinal tract^24,25^. Gut microbiota dysbiosis has been linked to metabolic disorder and impaired amino acid metabolism^26^. Downregulated amino acids might further change GLP-1 release and impact host metabolism through amino acids sensing perturbation^27^. Amino acids sensing signals contribute to GLP-1 release from enteroendocrine L cells^28^. Moreover, amino acids administered by oral ingestion as well as intravenous infusion have been reported to promote GLP-1 release to regulate glucose metabolism disorder^29^, such as tryptophan^30^, glutamine^31^, and L-arginine^32,33^.

GLP-1 expressing L cells with the highest density are in the distal small bowel and colon^34^. Panaro *et al*. further reported that Gcg and endocrine L cells in the distal gut were rapid responders to GLP-1 secretagogues^35^. GLP-1 has multiple metabolic effects, such as glucose-dependent stimulation of insulin secretion, suppression of pancreatic glucagon secretion, decrease of gastric emptying, and inhibition of food intake^36–38^. Decreased GLP-1 induced by nisin could further impact the secretion of glucoregulatory hormone, namely glucagon and insulin, thus impacting glucose metabolism. The counterregulatory mechanism between the two hormones effectively maintains euglycemia under variable nutritional and metabolic conditions^39^. Disordered glucagon to insulin ratio plays vital roles in initiating and maintaining pathological hyperglycemic states^40^. Glucagon that stimulates glucose output was significantly increased in nisin-treated mice. Nisin also increased insulin secretion, which was consistent with the previous study that glucagon can stimulate insulin release through direct and indirect means^41^. Glucoregulatory hormones secretion perturbation might partially mediate nisin induced glucose metabolism disorder. And whether insulin resistance mediated this effect needs to be further explored.

In conclusion, APs, but not all, evaluated in this study induced glucose intolerance and perturbed gut microbiota independently of the synthetic or biogenic origin. What calls for special attention is that the biogenic AP nisin induced the most significant effects. Although quite promising, caution is warranted when considering biogenic APs as more natural alternatives for synthetic ones.

## Methods

### Animals and treatments

The protocols for animal experiments were approved by the Ethical Committee of Experimental Animal Care of Nanchang University (approval number: 0064257). Wild-type C57BL/6 J male mice (8–10 weeks of age) were purchased from Hunan Silaike Laboratory Animal Co., Ltd [Changsha, China, license numbers: SCXK (XIANG) 2019-0004]. After 1 week of adaption, the mice were weighed and randomized into control and different APs-treated groups (four mice/cage, three cages/group) (Fig. S1): (1) Control group: mice were fed with normal diet (LAD3001M; Trophic Animal Feed High-Tech Co., Ltd., Nantong, China) for 8 weeks. (2) APs-treated groups: mice were fed with normal diet (LAD3001M) supplemented with various APs at three times the ADI level (Trophic Animal Feed High-Tech Co., Ltd., Nantong, China) for 8 weeks. APs used in this study were listed in Table S1. The mice were fed with deionized water ad libitum and were kept in individually ventilated cage (325 × 210 × 180 mm) systems (Suzhou Fengshi Laboratory Animal Equipment Co., Ltd, Suzhou, China) at 20 °C ± 2 °C and 50 to 70% relative humidity with a 12:12-h light-dark cycle. Food intake and body weight changes of mice were also recorded. APs had no significant effects on the eating habits and body weight of healthy mice after 8-week treatment (Fig. S2A, B).

### Oral glucose tolerance test

Mice were placed in a clean cage and fasted overnight for 16 h with free access to water^42,43^. Glucose tolerance testing was performed using oral gavage of 2 g/kg body mass glucose. Blood samples were collected by tail-tip bleeding 0, 15, 30, 60, 90 and 120 min later. The blood glucose concentrations were measured using a glucometer (Bayer, Leverkusen, Germany).

### Enzyme-linked immunosorbent assay (ELISA)

Glucagon and insulin levels in serum that collected on the final day of treatment were measured by ELISA using commercially available kits as manufacturer’s instructions (Cusabio, Wuhan, China).

### Quantitative PCR (qPCR)

Total RNA was extracted from colonic samples homogenized in RNAiso Plus (Takara, Beijing, China). One microgram of RNA was used to generate cDNA with a PrimeScript ™ RT reagent Kit (Takara, Beijing, China). The resulting cDNA was then subjected to qPCR using TB Green® Premix Ex Taq™ II (Takara, Beijing, China) on a CFX Connect real-time PCR detection system (Bio-Rad Laboratories, Singapore). The relative mRNA expression levels were determined with the 2^−ΔΔCt^ method with GAPDH as the internal reference control as previously described^14,44^. Results were presented as relative quantity. Primer sequences were as follows: Gcg forward: GCCACTCACAGGGCACATTC, Gcg reverse: CAGAGAAGGAGCCATCAGCG. Pcsk1 forward: AGTTGGAGGCATAAGAATGCTG, Pcsk1 reverse: GCCTTCTGGGCTAGTCTGC. Pcsk2 forward: GTTCCGGAGAGATTCCATTGT, Pcsk2 reverse: CAGGTAGCGGACGAAGTTT. GAPDH forward: TTCAGCTCTGGGATGACCTT, GAPDH reverse: TGCCACTCAGAAGACTGTGG.

### DNA extraction, 16S rDNA sequencing and bioinformatics analysis

The genomic DNA was extracted from feces samples using magnetic soil and stool DNA kit (TIANGEN, Beijing, China) optimized for an automated platform on the Biomek 4000 workstation (Beckman Coulter, Inc., Brea, CA, USA). The V4 region of bacterial 16S rDNA was selected to analyze using Illumina MiSeq sequencing, which was performed by a commercial company (Novogene, Co., Ltd., Beijing, China). Sequencing libraries were generated using TruSeq® DNA PCR-Free Sample Preparation Kit (Illumina, San Diego, CA, USA) following manufacturer’s recommendations and index codes were added. The library quality was assessed on the Qubit@ 2.0 Fluorometer (Thermo Scientific, MA, USA) and Agilent Bioanalyzer 2100 system (Agilent Technologies, CA, USA). Then, the library was sequenced on an Illumina NovaSeq platform (Illumina, San Diego, USA) and 250 bp paired-end reads were generated. Analysis of the 16S rDNA gene sequences were performed with Quantitative Insights into Microbial Ecology version 2 (QIIME2, version 2021.4)^45^. Deblur was used to generate high quality amplicon sequence variant (ASV) data^46^. The resulting ASV sequences were assigned to Greengenes database using q2-feature-classifier plugin^47^. Alpha diversity and beta diversity were analyzed using q2-diversity plugin. Principal coordinates analysis (PCoA) plots were visualized via emperor plugin^48^. Group-level differences were analyzed by PERMANOVA. For each PERMANOVA, false discovery rate (FDR) corrected *p*-values were calculated to control for multiple comparisons. *P*-values were considered statistically significant at FDR < 0.05.

### Targeted metabolomics analysis

Targeted metabolomics of feces samples were performed by Metabo-Profile (Shanghai, China). The sample preparation procedures were according to the previously published methods with minor modifications^49^. Briefly, 5 mg of lyophilized feces were homogenized with zirconium oxide beads and methanol containing internal standard to extract the metabolites. The resulting supernatants were subjected to derivatization with 3-nitrophenylhydrazineand N-(3-(dimethylamino)propyl)-N′-ethylcarbodiimide. HCl (Sigma-Aldrich, St. Louis, MO, USA). Subsequently, the derivatized samples were analyzed by ultra-performance liquid chromatography coupled to tandem mass spectrometry (UPLC-MS/MS) system (ACQUITY UPLC-Xevo TQ-S, Waters Corp., Milford, MA, USA). All of the standards were obtained from Sigma-Aldrich (St. Louis, MO, USA), Steraloids Inc. (Newport, RI, USA) and TRC Chemicals (Toronto, ON, Canada). The quality control samples were prepared following the same procedures as the test samples and were injected at regular intervals to ensure the instrument system stability. The raw data files generated by UPLC-MS/MS were processed using the Targeted Metabolome Batch Quantification software (v1.0, HMI, Shenzhen, Guangdong, China) to perform peak integration, calibration, and quantitation for each metabolite^49^. The iMAP platform (v1.0, Metabo-Profile, Shanghai, China) was used for statistical analysis, including PCA, OPLS-DA and pathway analysis.

### Statistical analysis

Unless otherwise stated in individual method section above, all the statistical analysis was performed with GraphPad Prism 8.21 software (La Jolla, CA, USA). Comparisons between two groups were assessed by two-tailed Student’s *t*-test. One-way ANOVA followed by Dunnett post hoc test was used to compare mean difference for more than two groups and adjusted *p*-values were calculated. *P*-value < 0.05 was considered to be statistically significant difference in all statistical analyses. Numbers of animals (*n*) used for individual experiments and details of the statistical tests used were indicated in the respective figure legends.

## Supplementary information

Supplementary Information

## Data Availability

The datasets presented in this study can be found in online repositories. All the 16S rDNA sequencing data can be found in the National Genomics Data Center (NGDC) BioProject database with the accession number PRJCA007659.
