# Supplementary material for: Systematic evaluation of antimicrobial food preservatives on glucose metabolism and gut microbiota in healthy mice
Source: NPJ Sci Food. 2022 Sep 13;6:42. doi: 10.1038/s41538-022-00158-y (PMC9470552; doi:10.1038/s41538-022-00158-y)
Supplement: Supplementary file 1 — Supplementary Information [file 41538_2022_158_MOESM1_ESM.pdf]

Systematic evaluation of antimicrobial food preservatives on glucose metabolism and gut microbiota in healthy mice

Ping Li, Ming Li, Tao Wu, Ying Song, Yan Li, Xiaochang Huang, Hui Lu*, Zhenjiang Zech Xu*

State Key Laboratory of Food Science and Technology, Nanchang University, No. 235 Nanjing East Road, Nanchang 330047, China

*Corresponding author:

Hui Lu, State Key Laboratory of Food Science and Technology, Nanchang University, No. 235 Nanjing East Road, Nanchang 330047, China. E-mail: luhui555simm@foxmail.com

Zhenjiang Zech Xu, State Key Laboratory of Food Science and Technology, Nanchang University, No. 235 Nanjing East Road, Nanchang 330047, China. E-mail address: zhenjiang.xu@ncu.edu.cn (Z. Xu)

Supplementary Figures:

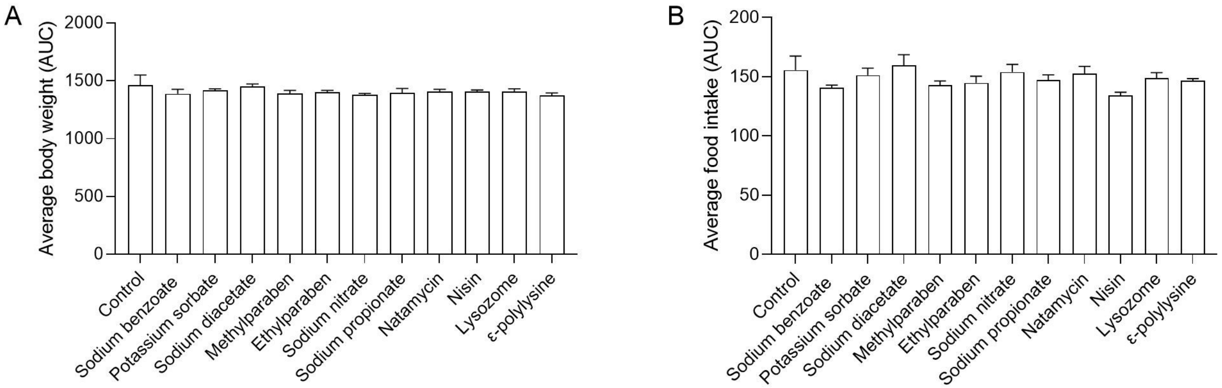

Supplementary figure 1. Average food intake and average body weight of different groups. A, Area under the eight-week body weight curve. B, Area under the eight-week food intake curve. The values are expressed as the means ± S.E.M., n = 9 per group.

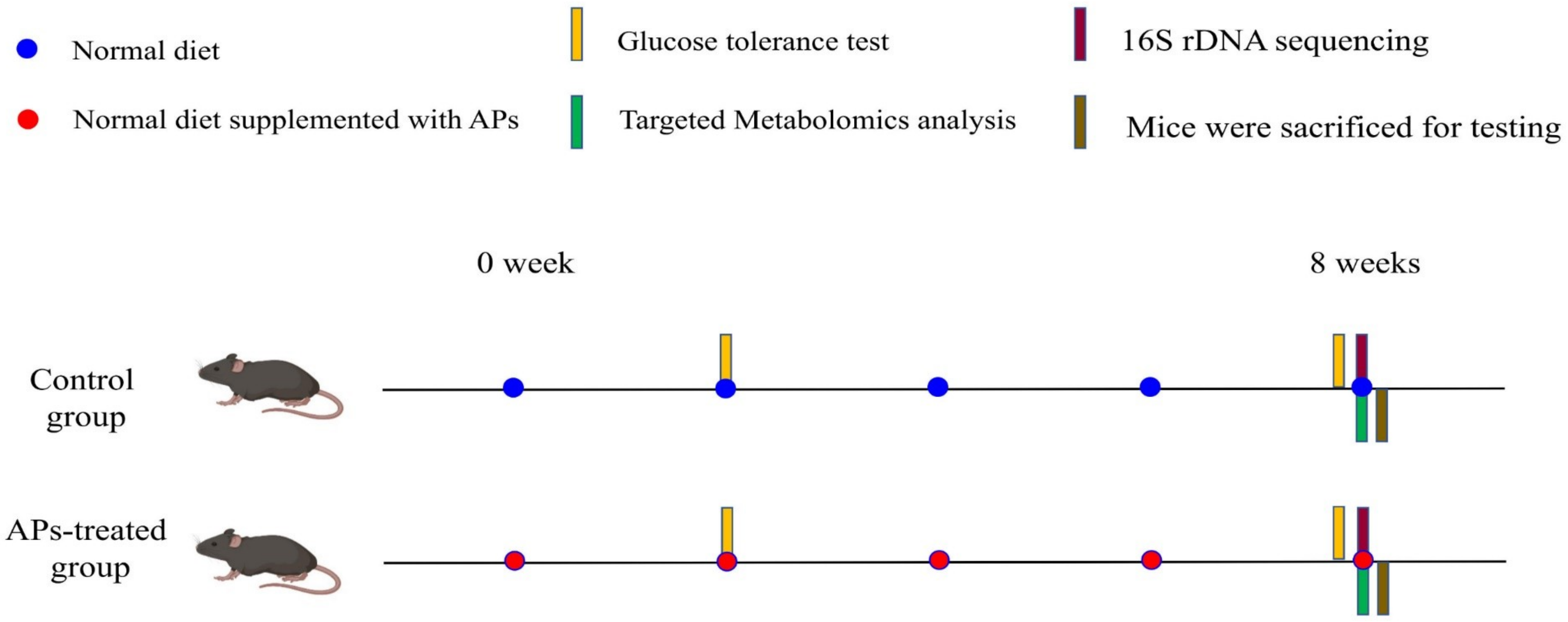

Supplementary figure 2. Experimental scheme. After 1 week of acclimation, the mice were randomized into control and APs-treated groups and treated for 8 weeks.

Supplementary Table:

Supplementary table 1. List of the eleven antimicrobial preservatives used.

Antimicrobial preservative	Origin (synthetic or biogenic)	E No[1]	CAS No	Molecular Weight (g/mol)	ADI[2] (mg/kg bodyweight)	Source	Purity	Calculation formula for supplementation
Sodium benzoate	synthetic	211	532-32-1	144.10	0-5	Sigma-Aldrich	99%	15 mg kg^{-1} day^{-1} * average mouse weight 0.03 kg * 56 days
Potassium sorbate	synthetic	202	24634-61-5	150.22	0-25	Sigma-Aldrich	99%	75 mg kg^{-1} day^{-1} * average mouse weight 0.03 kg * 56 days
Sodium diacetate	synthetic	262(ii)	126-96-5	142.09	0-15	Yuanye Bio, China	99%	45 mg kg^{-1} day^{-1} * average mouse weight 0.03 kg * 56 days
Methylparaben	synthetic	218	99-76-3	152.15	0-10	Sigma-Aldrich	99%	30 mg kg^{-1} day^{-1} * average

								mouse weight 0.03 kg * 56 days
Ethylparaben	synthetic	214	120-47-8	166.17	0-10	Sigma-Aldrich	99%	30 mg kg^{-1} day^{-1} * average mouse weight 0.03 kg * 56 days
Sodium nitrate	synthetic	251	7631-99-4	84.99	0-3.7	Sigma-Aldrich	99%	11 mg kg^{-1} day^{-1} * average mouse weight 0.03 kg * 56 days
Sodium propionate	synthetic	281	137-40-6	96.06	Not limited; Max level: 3000 mg/kg-GMP[3]	Sigma-Aldrich	99%	9 g kg^{-1} * average food intake 3 g day^{-1}* 56 days

Natamycin	biogenic	235	7681-93-8	665.74	0-0.3	Rhawn, China	98%	0.9 mg kg^{-1} day^{-1} * average mouse weight 0.03 kg * 56 days
Nisin	biogenic	234	1414-45-5	3354.07	0–2	Meilunbio, China	activity: ⩾900IU/mg	6 mg kg^{-1} day^{-1} * average mouse weight 0.03 kg * 56 days
Lysozyme	biogenic	1105	12650-88-3	about 14,000	Not specified; Max level: 500 mg/kg-GMP[3]	Solarbio, China	activity: 20000U/mg	1.5 g kg^{-1} * average food intake 3 g day^{-1} * 56 days
ε-polylysine	biogenic	NA[5]	28211-04-3	3800.00	FDA: 50 mg/kg[4]	Yuanye Bio, China	95%	0.15 g kg^{-1} * average food intake 3 g day^{-1} * 56 days

[1]Acceptable daily intake (ADI): The amount of an additive in food that could be ingested orally on a daily basis over a lifetime without an appreciable health risk.

[2]In the European Union, all food additives are labeled with the letter "E" and a specific number.

[3]Codex General Standard for Food Additives online database.

[4] Food and Drug Administration (FDA) approved the use of ε-polylysine in cooked or sushi rice at levels up to 50 mg/kg.

[5]NA: not applicable.
